# Biological Age and Psychological Distress: Health Disparities Between Midlife Jewish and Arab Adults

**DOI:** 10.1093/geroni/igaf122.962

**Published:** 2025-12-31

**Authors:** Khalil Iktilat, Merav Asher, Roy Tzemah-Shahar, Maayan Agmon

**Affiliations:** Tel Aviv University, Tel Aviv, Tel Aviv, Israel; University of Haifa, Haifa, HaZafon, Israel; University of Haifa, Haifa, HaZafon, Israel; University of Haifa, Haifa, HaZafon, Israel

## Abstract

Health disparities between Jewish and Arab populations in Israel are well-documented, particularly in contexts of chronic diseases, aging, and socioeconomic conditions. These gaps are widening and could be related to increasing violence in Arab society and the associated increases in stress. However, most research on health disparities focuses on older adults who already have multiple diagnoses, overlooking midlife individuals who can provide valuable insights into the aging process and the underlying patterns of these disparities. This study examines middle-aged individuals, using Biological Age (BA) as an objective health marker and considering the role of psychological distress in these two populations. This cross-sectional study included 221 participants (age 43.8±1.6, 57% female, 112 Jewish, 109 Arab). BA was estimated using the Klemera-Doubal algorithm. Executive function was assessed using the Trail Making Test (TMT) and Stroop Test, psychological distress via the six-item Kessler Scale (K6), and socioeconomic status based on education and income levels. No significant difference in chronological age between groups were observed; however, BA was significantly higher among Arabs (p < 0.001), suggesting accelerated aging. Additionally, Arabs exhibited higher psychological distress (p < 0.001), lower socioeconomic status (p < 0.01), and poorer executive function (p < 0.001) than Jews. These findings highlight the need for early intervention to address disparities before they escalate. Elevated BA and psychological distress among Arabs underscore the urgent need for action to reduce violence and address socioeconomic and mental health inequalities, which may help reduce premature aging and improve health equity in Israel.

